# Editorial: Machine Learning in Action: Stroke Diagnosis and Outcome Prediction

**DOI:** 10.3389/fneur.2022.984467

**Published:** 2022-07-20

**Authors:** Vida Abedi, Yuki Kawamura, Jiang Li, Thanh G. Phan, Ramin Zand

**Affiliations:** ^1^Department of Public Health Sciences, College of Medicine, The Pennsylvania State University, Hershey, PA, United States; ^2^Department of Medicine, University of Cambridge, Cambridge, United Kingdom; ^3^Department of Molecular and Functional Genomics, Weis Center for Research, Geisinger Health System, Danville, PA, United States; ^4^Stroke and Aging Research Group, Clinical Trials, Imaging and Informatics Division, School of Clinical Sciences at Monash Health, Melbourne, VIC, Australia; ^5^Department of Neurology, Monash Health, Melbourne, VIC, Australia; ^6^Department of Neurology, College of Medicine, The Pennsylvania State University, Hershey, PA, United States

**Keywords:** machine learning, artificial intelligence, stroke, cerebrovascular events, electronic health records (EHR), electronic medical records (EMR), deep learning, natural language processing

Machine learning—the ability of computers to “learn” to perform a task rather than being explicitly programmed for the purpose—has seen significant developments in recent years. Biomedical research is no exception to its far-reaching impact and has seen more than a ten-fold increase in the number of publications related to machine learning in the last decade (1). In this Research Topic, we present recent advances in developing machine learning algorithms in the context of cerebrovascular diseases to highlight promising approaches that represent various areas of potential clinical utility in stroke care. The focus is on applications with high clinical value and a solid technical foundation.

Deployment of machine learning algorithms in the clinic principally involves four stages of the care workflow: primary prevention, acute-phase treatment, post-diagnosis prediction, and secondary prevention (2). Primary prevention includes personalized or stratified patient risk prediction and identification of gaps in care, whereas integration into acute phase treatment aims to aid physician diagnosis and referrals. Machine learning algorithms for post-diagnosis and secondary prediction can provide predicted outcomes that allow the identification of patients who would be responsive to treatment or require careful monitoring due to a higher risk of recurrent disease. Together, machine learning algorithms can aid clinical decision-making in each step by providing recommendations and pointing to possible missed cases for critical conditions. As suggested by Mainali et al., machine learning algorithms can have particular utility in alleviating two of the clinical challenges of stroke: the time-sensitive nature of the acute-phase treatment and the difficulty of predicting outcomes, especially in the acute phase. Given these potential benefits, calibrating the algorithms to prevent excessive alerts and supporting physician autonomy through careful assessment of human-computer interaction is key to maximizing adoption (3).

Electronic health records (EHR) are one of the principal sources of standardized clinical information on a patient and can serve as a valuable starting point for algorithm development. The results of Rana et al. are encouraging, demonstrating that models trained on EHR data outperformed models trained on a limited number of features clinically associated with stroke, confirming the benefits of additional information obtained by data extraction from EHR. Using EHR data, Darabi et al. compared the performance of multiple machine learning models in predicting 30-day hospital readmission. Their models improved upon previous predictive models based on logistic regression and provided promising results that could direct targeted intervention for high-risk patients. Notably, features that their best predictive model indicated as being key predictors of 30-day readmission agree with results from independent studies (4, 5) and clinical intuition, underscoring the interpretability of their model.

Complementation of EHR data with additional modalities of clinical investigations holds promise in further improving prediction accuracy. Herein, Lineback et al. employ Natural Language Processing (NLP) to glean freeform textual data. In contrast, Rajashekar et al. combines MRI and CT imaging data to improve prediction models trained solely on EHR data. Multimodal approaches can require more sophisticated models to extract information from various data types but more closely approximate decision-making by physicians and better integrate multifaceted information collected *via* clinical investigations and examinations.

Imaging is a rich source of information. Imaging has critical clinical relevance in neurology and a high affinity for sophisticated deep learning models, such as convolutional neural networks. Indeed, many of the recent advances in machine learning in healthcare have centered on image analysis, including the use of retinal images for cardiometabolic disease prediction (6–9) and analysis of histopathological slides (10–15). Models focus on cerebrovascular disease, however, have been comparatively scant. McLouth et al. validate the performance of a commercially available deep learning software in assessing intracranial hemorrhage and large vessel occlusion using CT images. Implementing analysis software within the imaging workflow can provide venues where machine learning algorithms can seamlessly integrate into clinical decision-making. Furthermore, incorporating features from MRI scans, such as in the study by Xiao et al. predicting hypoperfusion in ischemic stroke patients, could define a clinically relevant threshold that directs decision-making in a facile manner. Integration of images in machine learning algorithms provides several benefits, including higher accuracy of diagnosis and improved objectivity compared to physical examinations. Given that imaging is routinely performed for stroke patients and is uniquely capable of providing functionally relevant anatomical information, image analysis models are promising candidates for clinical deployment in stroke care.

Machine learning can be an invaluable asset, especially in cases where diagnosis requires extensive examination or training or when the diagnosis is based on subtle features and are thus inherently prone to misdiagnosis. The algorithms described by Kim et al. to identify acute central dizziness and by Lin et al. to identify mild stroke patients at risk of disability exemplify the possibilities—supporting physicians in making challenging clinical decisions. Both models closely approximate or outperform existing risk scores without requiring extensive neurological examinations, allowing more patients to be screened and thus reducing the chances of a deteriorating patient escaping notice.

While machine learning holds promises, several challenges persist in implementing these technologies in healthcare. First, technical limitations can stem from the type and quality of the datasets available. EHR data can often be poorly standardized and sparse, posing problems in model generalizability. Investigators such as Rana et al. and Darabi et al. have only used administrative data from EHR with additional clinical variables such as NIHSS. By contrast, mining the free text in the patient chart (such as provider note, triage notes, discharge note, etc.) pose significant challenges. The free text is written by multiple clinicians, often with successive clinicians copying and pasting the written comments by the previous clinicians (16) in addition to auto-generated text that populate the patient chart. In addition, the tabular nature of clinical data extracted from EHR can often pose a difficulty even for advanced deep learning modalities, which often fail to surpass performances on simpler tree-based architectures (17). However, performance can be improved by extensive regularization (18). Sophisticated machine learning algorithms have had better success when applied to image datasets; however, even these complex deep learning algorithms can suffer from confounding factors, partially due to variation amongst institutions. Indeed, a recent study demonstrated that deep neural nets trained to predict SARS-CoV2 infection from X-ray images tend to select confounding “shortcuts” over signals in generating predictions (19). Attributes of datasets can limit the accuracy and generalizability of models, especially for external cohorts with different demographics and dataset characteristics. The development of standardized data protocols can aid the implementation of machine learning models that are more accurate and generalizable across multiple institutions. In addition to curating better datasets, models can also be adjusted for better generalizability; fine-tuning of pre-trained algorithms *via* transfer learning using site-specific data achieved superior results for external cohorts (20), and continuous domain adaptation has been explored to tackle temporal drifts in data (21, 22). It is essential to take all possible precautions to ensure that the machine learning algorithms provide reliable, relevant, and interpretable results free from systemic biases. To achieve that, care must be taken to minimize confounding variations in the datasets that might affect generalizability and ensure fine-tuning approaches are integrated to allow the models to more closely approximate results for the underlying patient distribution.

Secondly, more complicated machine learning models can often be challenging to interpret, hindering the translation from prognosis to patient management. High-performing “black box” models lacking interpretability are of limited use in the clinic as they do little to inform physicians of actionable points. In particular, identifying modifiable risk factors is essential in the primary and secondary prevention of cerebrovascular events. To this end, Cui et al. used feature importance metrics to rank specific features mainly associated with predictive capability in each machine learning model. Analyses of feature importance could prove helpful in guiding intervention, especially if a factor is consistently listed as important across multiple models. For image analysis models, localization maps generated by methods such as Grad-CAM (23) could provide a limited level of interpretability. Separating interpretation from the prediction modeling to provide more flexibility is a strategy that has been getting more traction in recent years. Still, the usefulness of the algorithms can be diminished by confounding “shortcuts,” as mentioned earlier. Since model depth is generally associated with better predictive capability, efforts must be made to create models that predict and inform. Desirable models should also consider workflow disruption or the possibility of causing “alert fatigue” before planning for implementation. Designing and training models so that interpretable features can be gleaned from model parameters and incorporating feedback from healthcare providers can improve the interpretability of models. In this respect, theoretical advances in model architecture and interpretation, combined with enhancing training data robustness, could prove fruitful.

Finally, ethical considerations must not be ignored. Model predictions can often be influenced by the socioeconomic, racial, and gender composition of the training datasets, the awareness of which is necessary to mitigate potential biases in models. For example, machine learning models were found to consistently underdiagnose patients in disadvantaged populations across three large chest X-ray datasets, especially where a patient was a member of more than one underserved group (24). The precedent of undertreatment in disadvantaged populations can further exacerbate biases by making it less likely for the algorithm to recommend treatment for members of the underprivileged sub-group of the population if similar patients were not provided treatment in the past. The performance of machine learning models must thus be thoroughly evaluated in different cohorts to assess the presence of systematic bias, which must be rectified before deployment. Further, while it is often possible to impute information that a patient declined to provide (e.g., smoking, HIV status, etc.), doing so can have ethical implications (25). Implementing machine learning algorithms in the clinic should proceed with special care to avoid unwittingly perpetuating health care inequalities in the training cohort. Finally, it is essential to reflect that algorithms are and will continue to be part of our medical system, including our medical education system. Thus, as a two-way street, we have to consider how such recommendations influence physicians' decisions and how this decision-making process potentially shifts with continued interaction.

In conclusion, recent developments in machine learning present ample opportunities for automated models that guide clinical decision-making and improve patient outcomes. The studies included herein represent selections of advances employing machine learning in various contexts in stroke care in our collective efforts to promote improved patient health through effective prevention, diagnosis, and intervention.

## Author contributions

All authors listed have made a substantial, direct, and intellectual contribution to the work and approved it for publication.

## Conflict of interest

The authors declare that the research was conducted in the absence of any commercial or financial relationships that could be construed as a potential conflict of interest. The handling editor J-CB declared a shared affiliation with the author YK at the time of review.

## Publisher's note

All claims expressed in this article are solely those of the authors and do not necessarily represent those of their affiliated organizations, or those of the publisher, the editors and the reviewers. Any product that may be evaluated in this article, or claim that may be made by its manufacturer, is not guaranteed or endorsed by the publisher.
